# Biochemical parameters, dynamic tensiometry and circulating nucleic acids for cattle blood analysis: a review

**DOI:** 10.7717/peerj.8997

**Published:** 2020-05-22

**Authors:** Sergei Yu. Zaitsev, Nadezhda V. Bogolyubova, Xuying Zhang, Bertram Brenig

**Affiliations:** 1 Department of Physiology and Biochemistry of Farm Animals, Federal Science Center for Animal Husbandry Named After Academy Member L.K. Ernst, Podolsk, Moscow Region, Russian Federation; 2 Institute of Veterinary Medicine, University of Göttingen, Göttingen, Germany

**Keywords:** Blood, Biochemical parameters, Circulating nucleic acids, Tensiometry

## Abstract

The animal’s blood is the most complicated and important biological liquid for veterinary medicine. In addition to standard methods that are always in use, recent technologies such as dynamic tensiometry (DT) of blood serum and PCR analysis of particular markers are in progress. The standard and modern biochemical tests are commonly used for general screening and, finally, complete diagnosis of animal health. Interpretation of major biochemical parameters is similar across animal species, but there are a few peculiarities in each case, especially well-known for cattle. The following directions are discussed here: hematological indicators; “total protein” and its fractions; some enzymes; major low-molecular metabolites (glucose, lipids, bilirubin, etc.); cations and anions. As example, the numerous correlations between DT data and biochemical parameters of cattle serum have been obtained and discussed. Changes in the cell-free nucleic acids (cfDNA) circulating in the blood have been studied and analyzed in a variety of conditions; for example, pregnancy, infectious and chronic diseases, and cancer. CfDNA can easily be detected using standard molecular biological techniques like DNA amplification and next-generation sequencing. The application of digital PCR even allows exact quantification of copy number variations which are for example important in prenatal diagnosis of chromosomal aberrations.

## Introduction

Biological liquids (bioliquids) of animals and humans (blood, lymph, milk, etc.) contain cells, as well as many biopolymers and low-molecular surfactants (peptides, proteins, oligo- and polynucleotides, lipids, lipoproteins, surface inactive substances) (Harris, 1991; Schaller et al., 2008; Zaitsev & Konopatov, 2005; Basten, 2019). These substances can be analyzed by standard and methods such as dynamic tensiometry (DT) or polymerase chain reaction (PCR) followed by next-generation sequencing in the case of nucleic acids (Chitty & Lo, 2015).

DT is an “integrative” method studying the adsorption of biopolymers and low-molecular surfactants at the interfaces of the bubbles produced (by special device) in serum samples (Vozianov et al., 1999; Kazakov et al., 2000; Miller et al., 2001; Zaitsev et al., 2004; Khilko, 2014). Serum DT data of healthy people of various age and sex as well as for people having nephritis, obstructive bronchitis, autoimmune diseases such as systemic lupus erythematosus, diabetes mellitus, rheumatoid arthritis and other illnesses were defined already for a few decades (Vozianov et al., 1999; Kazakov et al., 2000; Spengler, 2001; Khilko, 2014). In contrast, the dependance of the DT parameters (complete quantitative data set) of the serum of some animals have been obtained recently by our group of researchers (Milayova et al., 2010; Zarudnaya et al., 2010; Zaitsev et al., 2011a; Zaitsev, 2016, 2018). The authors revealed the correlations between dynamic tensiometry and biochemical parameters of cattle serum that can simplify the procedure and to speed up the final diagnosis decision (Zaitsev, Maksimov & Bardyukova, 2008; Zaitsev, 2016; Voronina, 2017). The obtained DT and correlation data are included in the expanded database with the biochemical parameters of cattle serum and will be useful for animal health monitoring, quality assessment of the dairy and meat products. The strong correlations (no matter positive or negative they are) of DT with the biochemical parameters of cattle serum are especially important and have advantages to be included in the database in contrast to some middle or weak correlations of different types.

It is obvious that the importance of the data is increasing by joint consideration of the biochemical and DT parameters, as well as the circulating nucleic acids for large animal blood analysis that will discussed in this review. Special attention must be devoted to the highly productive cows which usually have relatively high risk of various diseases during lactation period (Constable, Trefz & Stämpfli, 2019; Kaneko, Harvey & Bruss, 2008; Kharitonov, 2011; Maximov et al., 2012).

The major biochemical parameters of the animal serum will be described in details here, whereas some other important components such as red blood cell (RBC) or white blood cell (WBC) counts will be only slightly mentioned in this review.

## Survey Methodology

This literature review examined the peer-reviewed and gray literature on biochemical, physical chemical and molecular biology methods for animal blood plasma and serum analysis. We searched PubMed, Google Scholar, ResearchGate and e-library to identify potential studies for inclusion on February 20, 2020. The keywords were the following: blood, biochemical parameters, dynamic tensiometry, circulating nucleic acids. We focused on manuscripts that defined modern approaches and conceptual issues, as well as applications of the abovementioned methods for animal medicine. We included also some (the most important to our review) studies that were not in English. This manuscript defines the biochemical, physical chemical and molecular biology methods for animal blood plasma and serum analysis.

### Standard biochemical analysis

It is known that standard biochemical tests do not always point to a diagnosis (Harris, 1991; Vozianov et al., 1999; Schaller et al., 2008; Basten, 2019), but still they can be helpful and commonly used for animals (Kaneko, Harvey & Bruss, 2008; Zaitsev, 2017; Voronina, 2017). These tests are helpful to speed up the final diagnosis decision, as well as for planning and control of “therapeutic” treatment (Zaitsev, Maksimov & Bardyukova, 2008; Zaitsev, 2016; Voronina, 2017). Of course, a particular diagnosis in the case of severу diseases usually needs specific tests (Zaitsev, Maksimov & Bardyukova, 2008; Constable, Trefz & Stämpfli, 2019; Kaneko, Harvey & Bruss, 2008). Interpretation of major biochemical parameters (MBP) usually is similar across major animal species, but there are a few peculiarities in each case, especially well-known for cattle.

#### Total protein content

Refractometry and spectrophotometry are the relatively simple and traditional tools to measure the “total protein” content (i.e., all proteins and popypeptides) in serum, whereas the numerious modifications of the electrophoretic technique are used to evaluate albumin and globulin fractions (Constable, Trefz & Stämpfli, 2019; Kaneko, Harvey & Bruss, 2008; Zaitsev, 2016). The particular data of the “total protein” content in cow’s blood are summarized in the Table 1. In general, the “total protein” content in cow’s blood is in the range of 60–75 g/l for adult animals and significantly increasing (up to 80–89 g/l) for lactating cows (Alberghina et al., 2011; Kholod & Ermolaev, 1988; Voronina, 2017; Zaitsev, 2016) depending on the age, breed, diet, particular lactation period, etc. (Table 1). Any changes of the blood protein values (significantly higher or lower the reference data, mentioned above) is an important key to dysproteinemia diagnosis (Maximov et al., 2012). Hyperproteinemia (increased “total protein” content) is due to dehydration or inflammation, whereas hypoproteinemia (decreased “total protein” content) is caused mainly by insufficient amount of adequate protein in nutrition for animals, diarrhea, etc. (Constable, Trefz & Stämpfli, 2019; Kaneko, Harvey & Bruss, 2008; Zaitsev, 2016). Anemia, problems in feeding of herds or specific animals, some their parasites should be evaluated first, then the problems with the gastrointestinal or urinary tracts should be suspected (Maximov et al., 2012).

**Table 1:** Total protein content and Albumin/Globulin (A/G) ratio in cow serum (mean ± SD).

Parameter value	Cattle breed	Farm place	References
Total protein content (mean ± SD)			
60–89 g/l	Simmental	Belorussia	Kholod & Ermolaev (1988)
69–74 (±2) g/l	Black & white	Moscow region, Russia	Voronina (2017)
66–74 (±2) g/l	Black & white before lactation	Moscow region, Russia	Zaitsev (2016)
80.4 ± 1.2 g/l	3 months lactation		Zarudnaya et al. (2010)
59-81 (±8) g/l	Holstein	Ontario, Canada	Lumsden, Mullen & Rowe (1980)
65.6 ± 1.32 g/l	Simmental	around Zagreb, Croatia	Žvorc et al. (2000)
66.7 ± 20.6 g/l	Holstein	3°27′N, 76°32′ W, Colombia	Campos et al. (2012)
57.7 ± 23.2 g/l	Hartón del Valle creole	Valle del Cauca department, 4°27′ N, 76°20′ W, Colombia	Campos et al. (2012)
	Slovak spotted	Košice, Slovak Republic	Tóthová, Mihajlovičová & Nagy (2018)
72.3 ± 4.4 g/l	ante partum (prepartal) period 1 week		
73.5 ± 4.2 g/l	post partum (postpartal) period 1 week		
77.6 ± 5.2 g/l	post partum (postpartal) period 3 weeks		
78.8 ± 4.3 g/l	post partum (postpartal) period 6 weeks		
67.54 ± 11.53 g/l	Modicana cattle	Sicily, Italy	Alberghina et al. (2011)
Albumin/globulin (A/G) ratio			
0.88 ± 0.43	Modicana cattle breed	Sicily, Italy	Alberghina et al. (2011)
0.80	Simmental breed	Belorussia	Kholod & Ermolaev (1988)
0.74–0.92	Black & white breed	Moscow region, Russia	Voronina (2017)
0.65–0.90	Black & white breed	Moscow region, Russia	Zaitsev (2016)
0.6–1.3	Holstein	Ontario, Canada	Lumsden, Mullen & Rowe (1980)
0.75–0.98	Brahman crossbreed cattle	Au Giang Province, Vietnam	Xuan, Loc & Ngu (2018)

#### Albumin to globulins ratio, fibginogen and “acute phase proteins”

It is obvious that serum is the “blood plasma after coagulation”, that is, without fibrinogen (Schaller et al., 2008; Zaitsev, 2016). That is why, a fibrinogen level is easy to estimate using the difference in the “total protein” values in plasma and serum of the same blood samples. There is not only increasing content of some usual proteins, such as fibrinogen, found by inflammation, but also appearance of special so-called “acute phase proteins” is observed (Constable, Trefz & Stämpfli, 2019; Maximov et al., 2012). Appearance of the “acute phase proteins”, such as C-reactive protein (CRP), cryoglobulins, etc., in blood can be considered as inflammation markers both for humans and animals (Kholod & Ermolaev, 1988; Maximov et al., 2012; Zaitsev & Konopatov, 2005; Zaitsev, 2017). The fibrinogen and globulin levels increased by inflammation for different times, but both are important to evaluate because WBC count level not always accurately points to inflammation for some animals. In general, for cattle a left shift in the complete blood cell count can occurs early and correlates with a degree of inflammation (Constable, Trefz & Stämpfli, 2019; Kaneko, Harvey & Bruss, 2008; Zaitsev, 2016).

The total values and content of the protein fractions are among the most important “integral” biochemical parameters for description of the methabolic activity not only in blood and other tissues, but in the animal organism as a whole. Depending on the age, lactation period, supplements to the basic diet, etc., the albumin/globulin (A/G) ratios of cow’s blood are summarized in the Table 2. In general, the A/G ratios of cow’s blood (Table 1) are in the range of 0.66–0.90 (Alberghina et al., 2011; Kholod & Ermolaev, 1988; Voronina, 2017; Zaitsev, 2016; Xuan, Loc & Ngu, 2018). So, the average value around 0.8 seems to be reasonable reference for cattle A/G ratio. The values of some important blood enzymes will be presented and discussed below.

**Table 2:** Aspartate transaminase (AST), alanine transaminase (ALT) and gamma-glutamyltransferase (GGT) contents in cow serum.

Parameter value	Cattle breed	Farm place	References
Aspartate transaminase (AST) content			
78–132 U/L	Data not available	Data not available	Kaneko, Harvey & Bruss (2008)
183–2667 nkat/L	Simmental breed	Belorussia	Kholod & Ermolaev (1988)
62–82 U/L	Black & white breed	Moscow region, Russia	Voronina (2017) and Zaitsev (2016)
24–45 U/L	Holstein	Ontario, Canada	Lumsden, Mullen & Rowe (1980)
19.3–37.7	Brahman crossbreed cattle	Au Giang Province, Vietnam	Xuan, Loc & Ngu (2018)
934–1417 nkat/L	Data not available	Kazan region, Russia	Hazipov & Askarova (2003)
Alanine transaminase (ALT) content			
11–40 U/L	Data not available	Data not available	Kaneko, Harvey & Bruss (2008)
22–1000 nkat/L	Simmental breed	Belorussia	Kholod & Ermolaev, 1988
62–82 U/L	Black & white breed	Moscow region, Russia	Voronina (2017) and Zaitsev (2016)
5–18 U/L	Holstein	Ontario, Canada	Lumsden, Mullen & Rowe (1980)
13.8–26.5 U/L	Brahman crossbreed cattle	Au Giang Province, Vietnam	Xuan, Loc & Ngu (2018)
450–700 nkat/L	Data not available	Kazan region, Russia	Hazipov & Askarova (2003)
Gamma-glutamyltransferase (GGT) content			
6.1–17.4 U/L	Data not available	Data not available	Kaneko, Harvey & Bruss (2008)
111.7–483.4 nkat/L	Simmental breed	Belorussia	Kholod & Ermolaev (1988)
28–44 U/L	Black & white breed	Moscow region, Russia	Voronina (2017) and Zaitsev (2016)
450–700 nkat/L	Data not available	Kazan region, Russia	Hazipov & Askarova (2003)

#### Serum enzymes

Enzyme blood tests start to be very popular nowadays (Constable, Trefz & Stämpfli, 2019; Kaneko, Harvey & Bruss, 2008; Zaitsev, 2016). The most important and useful enzymes in the case of animal blood tests are the following: lactate-dehydrogenase (LDH), aspartate transaminase (AST), alanine transaminase (ALT), gamma-glutamyltransferase (GGT), etc. (Schaller et al., 2008; Zaitsev, 2010; Zaitsev, 2016, 2017). It is important to highlight that their results should be “interpreted with caution”, because of the huge variation in the reference values even for healthy animals (Vozianov et al., 1999; Zaitsev, 2016; Voronina, 2017).

For example, normal AST values for cows are in the range of 78–132 U/L (Kaneko, Harvey & Bruss, 2008) or 183–2,667 nkat/L (Kholod & Ermolaev, 1988) depending on the age, sex, breed, feeding, etc. (Table 2). In any case the values about 19.3–37.7 U/L (Xuan, Loc & Ngu, 2018) looks strange, but may be explained by variety of supplements to the basic diet (grass, rice straw and rice bran) in the case of Brahman crossbreed cattle (Au Giang Province, Vietnam). In general, ALT values in cattle blood are lower (as compared to AST) and in the range of 11–40 U/L (Kaneko, Harvey & Bruss, 2008) or 450–700 nkat/L (Hazipov & Askarova, 2003) depending on the age, sex, breed, feeding, etc. (Table 2). Our data for adult cattle are the following: AST 62–82 U/L for cows or 67-98 U/L for bulls; ALT 32–36 U/L for cows or 23–29 U/L for bulls at standard farm feeding in Russia during last decades (Zaitsev, 2016, Voronina, 2017). These results are in a reasonable agreement with the majority of the obtained data (Constable, Trefz & Stämpfli, 2019; Kaneko, Harvey & Bruss, 2008; Xuan, Loc & Ngu, 2018), but differ to the data obtained by researchers from Belorussia (Kholod & Ermolaev, 1988) and Tatarstan (Hazipov & Askarova, 2003), which used another analytical approaches. Nevertheless, a pronounced increase in the AST or ALT values (in 4–8 times) is a clear evidence of heart (muscle) or liver disorder even before the clinical evidence. An increase in the ALT level is faster in the “light” cases, whereas, an increase in the AST level is faster in the much more serious cases (Zaitsev, 2016, Voronina, 2017).

In general, GGT values in cattle blood are in the range of 6.1–17.4 U/L (Kaneko, Harvey & Bruss, 2008) or 450–700 nkat/L (Hazipov & Askarova, 2003) depending on the age, sex, breed, feeding, etc. (Table 2). Our data for adult cattle are the following: 28–44 U/L for cows and 30–50 U/L for bulls at standard farm feeding in Russia (Zaitsev, 2016; Voronina, 2017). An increasing GGT content in blood can be connected with some liver diseases such as “cholestasis and hepatocellular membrane damage” in cattle (Constable et al., 2016; Zaitsev, 2017). It is important to highlight that the increased GGT values in blood observed much longer as compared to the increased values of ALT, ACT or some other enzymes. The elevated levels of the abovementioned enzymes in blood indicated that the liver should be further investigated (Maximov, Goudin & Lysov, 2010; Maximov et al., 2012; Zaitsev, 2017). The ALT or ACT isoenzyme profile can be especially useful for specific diagnosis, but it rather complicated and expansive for cattle and used mainly for dogs and cats (Zaitsev, Maksimov & Bardyukova, 2008). Any “malfunction” (mutation, overproduction, underproduction or deletion) of a single enzyme can also indicate a genetic disease. Thus, the general control of enzyme activity is essential for cattle diagnostics (Zaitsev & Konopatov, 2005; Zaitsev, 2016, 2017).

#### Low molecular serum components

The level of the hemoglobin degradation products, such as total bilirubin, is an important indicator for some pathological conditions (e.g., massive hemolysis of erythrocytes in malaria, obstruction of the bile ducts or other liver diseases) (Constable et al., 2016; Zaitsev, 2017). The reference values of the total bilirubin for cattle (summarized in the Table 3) are in the range of 0–16 µM/L (McSherry et al., 1984; Kholod & Ermolaev, 1988; Kaneko, Harvey & Bruss, 2008; Zaitsev, 2016). The enormous high upper limit of the total bilirubin values in cattle blood (up to 29.67 µM/L) published by Vietnam scientists (Xuan, Loc & Ngu, 2018) may be explained by variety of supplements to the basic diet (grass, rice straw and rice bran) in the case of Brahman crossbreed cattle (Au Giang Province, Vietnam) (Table 3). Moreover, cattle hyperbilirubinemia is well described in the paper of Canadian vets (McSherry et al., 1984). If the level of the hemoglobin degradation product (such as total bilirubin) is abnormal, then it is important to measure the values of the “indirect” (unconjugated) and “direct” (conjugated) bilirubin separately (McSherry et al., 1984; Zaitsev, 2016; Xuan, Loc & Ngu, 2018).

**Table 3:** Total bilirubin content in cow serum.

Parameter value	Cattle breed	Farm place	References
0.5–16 μM/L	Data not available	Data not available	Kaneko, Harvey & Bruss (2008)
3.42–10.26 μM/L	Simmental breed	Belorussia	Kholod & Ermolaev (1988)
4–10 μM/L	Black-&-white breed	Moscow region, Russia	Voronina (2017) and Zaitsev (2016)
0–8.6 μM/L	Holstein	Ontario, Canada	Lumsden, Mullen & Rowe (1980)
5.23–29.67 μM/L	Brahman crossbreed cattle	Au Giang Province, Vietnam	Xuan, Loc & Ngu (2018)
0–9 μM/L	Data not available	Data not available	McSherry et al. (1984)

The large deviations in the reference values of the total lipids, triglycerides, fatty acids, cholesterol and phospholipids are discussed in the following reviews and papers (Guédon et al., 1999; Adewuyi, Gruys & Van Eerdenburg, 2005; Zaitsev, 2016).

It is strange that enormous low values of the glucose (from 0.57 mM/L to 1.83 mM/L) in cattle blood published without discussions in the following paper (Xuan, Loc & Ngu, 2018) (Table 4). It may be explained by variety of supplements to the basic diet (grass, rice straw and rice bran) in the case of Brahman crossbreed cattle (Au Giang Province, Vietnam). In contrast, the reasonable glucose values in the cattle blood can be considered as reference: from 2.86 mM/L to 5.66 mM/L (Kholod & Ermolaev, 1988), 2.50–4.16 mM/L (Kaneko, Harvey & Bruss, 2008) (Table 4).

**Table 4:** Glucose content in cow serum.

Parameter value	Cattle breed	Farm place	References
2.50–4.16 mM/L	Data not available	Data not available	Kaneko, Harvey & Bruss (2008)
2.86–5.66 mM/L	Simmental breed	Belorussia	Kholod & Ermolaev (1988)
2.60–4.10 mM/L	Black-&-white breed	Moscow region, Russia	Voronina (2017) and Zaitsev (2016)
2.5–3.8 mM/L	Holstein	Ontario, Canada	Lumsden, Mullen & Rowe (1980)
0.57–1.83 mM/L	Brahman crossbreed cattle	Au Giang Province, Vietnam	Xuan, Loc & Ngu (2018)
0–9 μM/L	Data not available	Data not available	McSherry et al. (1984)

#### Major inorganic cations and anions

The reference values of the major cations (sodium, potassium, calcium, magnesium) and anions (chlorides, total bicarbonates or CO_2_ index, phosphates, etc.) are another essential part of the “serum chemistry profile” of any human or animal (Zaitsev & Konopatov, 2005; Zaitsev, 2016, 2017). The reference values of the major cations and anions for cattle blood are not always presented in the biochemical papers. That is why the authors just showed the most reasonable values here: 140–150 (Na^+^), 4.3–5.8 (K^+^), 2.2–2.8 (Ca^2+^), 0.8–1.2 (Mg^2+^), 105–120 (Cl^−^) mM/L (Kholod & Ermolaev, 1988; Zaitsev, 2016), 17–29 (HCO_3_^−^) mM/L (Kaneko, Harvey & Bruss, 2008; Zaitsev, 2016). Metabolic alkalosis (at high CO_2_ level), hypochloremia (low chlorides level) and hypokalemia (low potassium level) are the common abnormalities in adult cattle “with gastrointestinal disease” (Zaitsev & Konopatov, 2005; Zaitsev, 2016, 2017). Hyponatremia (low sodium level) and hypochloremia (low chlorides level) are usually occurs together with diarrhea. In the case of acidosis, a hyperkalemia (high potassium level) can be observed, but the blood potassium level is rarely increased essentially and rapidly (Zaitsev & Konopatov, 2005; Zaitsev, 2016, 2017). From one side, a little hypocalcemia (middle low potassium level) can be easily recognized during physical examination of sick cattle without additional biochemical tests, but on the other side, blood biochemistry analysis might be helpful in the most cases of the cation or anion problems (Zaitsev & Konopatov, 2005; Zaitsev, 2016, 2017). In this respect it is noteworthy that hypercalcemia is rare observed, even by general animal treatment using calcium (Zaitsev & Konopatov, 2005; Zaitsev, 2016, 2017). It is important to highlight that the abnormal electrolyte blood parameters are mainly caused by animal nutrition problems.

### Dynamic tensiometry analysis

Both biopolymers and low-molecular surfactants can be easily adsorbed at the liquid interfaces (such as “bioliquid-air”) with corresponding changes of surface tension (ST) values (Butt, Graf & Kappl, 2006; Kazakov et al., 2000; Khilko, 2014; Voronina, 2017; Zaitsev et al., 2004; Zaitsev, 2016, 2018). This process is considered to be one of the main mechanisms of the surfactant activity playing a significant role in numerous functions of animals (Miller & Fainerman, 1998; Zarudnaya et al., 2010; Zaitsev et al., 2011b; Zaitsev, 2010, 2016, 2018). It is important to highlight that changes of the concentration of biologically active substances (BAS) in biological liquids are influenced by particular periods, for example, growth, development, pregnancy, lactation, and adaptation to changing environmental conditions (stress). Thus, the major changes in BAS concentration (i.e., DT parameters of these liquids) can be expected during various illnesses or particular cases with deviation of physiologic-biochemical status.

#### The methods and equipment of the measurements of dynamic tensiometry parameters of biological liquids

There are numerious methods and devises for the measurements of dynamic tensiometry parameters of biological liquids (Miller & Fainerman, 1998; Hubbard, 2002; Somasundaran, 2015). The one of the most convenient for blood study is the tensiometer BPA-1P (so-called “Maximum Bubble Pressure Tensiometer”). The function principle of the tensiometer BPA-1P and its new generations is based on the maximum pressure measurements in the bubble method (Miller & Fainerman, 1998; Kazakov et al., 2000; ; Khilko, 2014; Voronina, 2017; Zaitsev et al., 2004, 2011a; Zaitsev, 2016, 2018).

**Figure 1:**
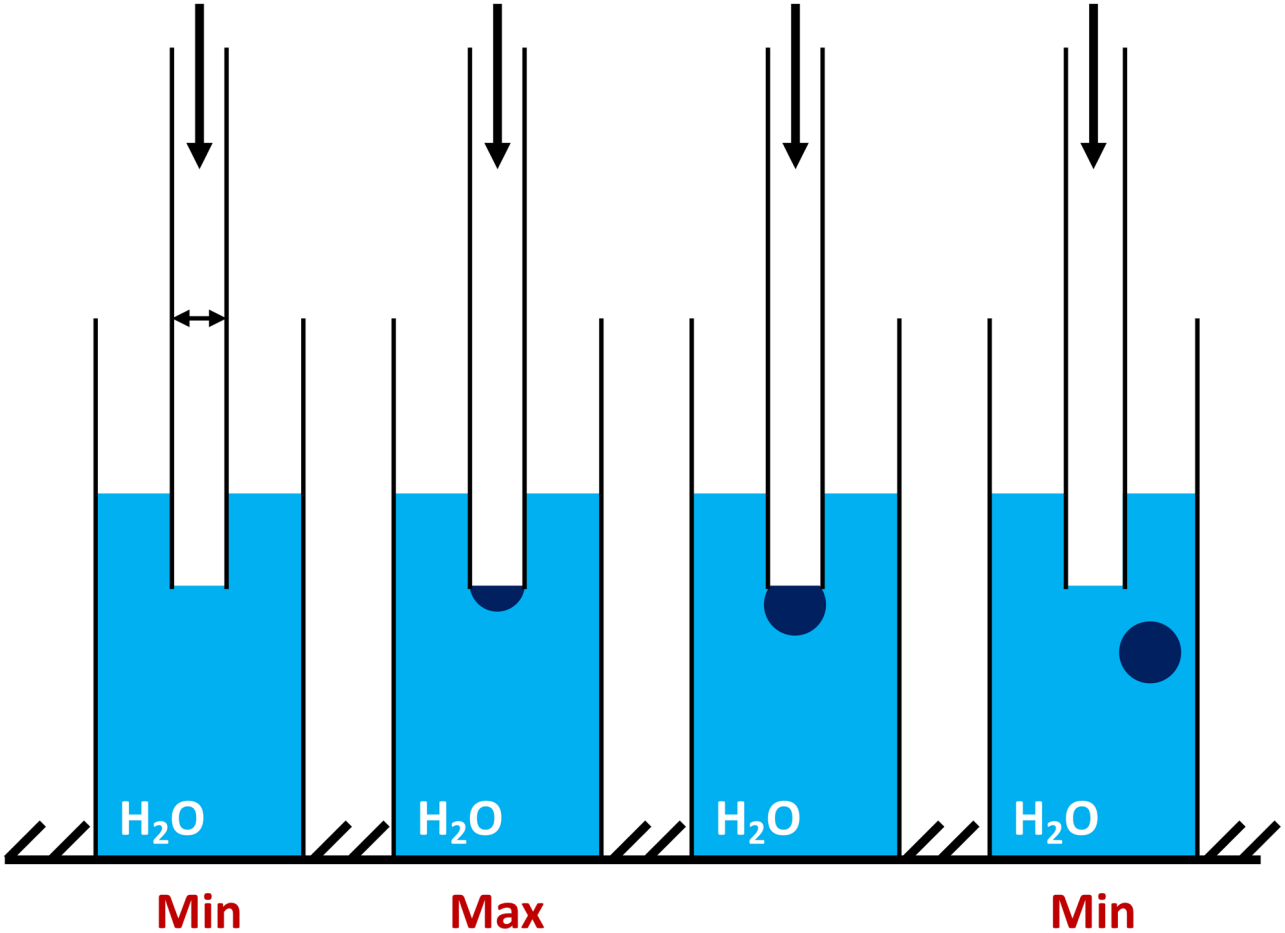
The principle concept of the maximum bubble pressure measurements by tensiometer BPA-1P (adapted from http://sinterface.de/). “Min” (at the left insert) is showing the initial pressure pulling down the water level in the capillary; “Max” (at the middle insert) is showing the moment when the bubble takes the form of a hemisphere capillary radius (equal to the radius of curvature) and pressure reaches the maximum value; “Min” (at the right insert) is showing the further bubble growth and avulsion.

The significant advantages of the BPA and relative devices are the following: a small volume of sample, high analysis speed, full automation of the measurement process, the computer processing of the information received (Zaitsev, 2016). The air from the compressor enters the capillary, which is lowered into the test liquid. The maximal pressure in the system is determined (Fig. 1) and used to calculate the surface tension (Zaitsev, 2016).

The pressure required for the separation of the air bubble from the capillary tip, that drops at the air-liquid interfaces, is directly proportional to the surface tension (σ). To overcome the wetting phenomenon in the capillary (dipped into liquid) an excessive air pressure is required. The maximum pressure that occurs during the formation of the air bubble during blowing depends on the capillary radius (Fig. 1 “min” at the left insert). At the moment when the bubble takes the form of a hemisphere capillary radius is equal to the radius of curvature and pressure reaches the maximum value (Fig. 1 “max” at the middle insert). With further bubble growth the curvature radius increases again, which reduces the pressure inside the bubble (Fig. 1 “min” at the right insert). The division of the interval between the bubbles into the so-called “dead time” and the surface “lifetime” is based on the existence of a critical point, depending on the air flow pressure (Zaitsev, 2016). Comparison of the data obtained by BPA-1P with those by other well-known methods (oscillating jet, drop volume, dynamic, capillary, etc.) (Miller & Fainerman, 1998; Kazakov et al., 2000; Khilko, 2014; Voronina, 2017; Zaitsev et al., 2004, 2011b; Zaitsev, 2016, 2018) showed a good agreement between the results.

**Figure 2:**
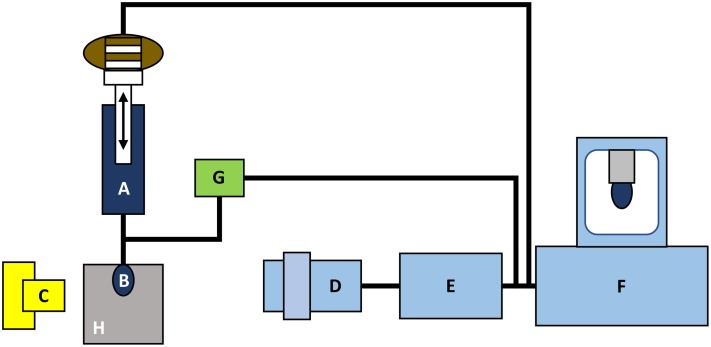
The principle concept of the DST measurements by tensiometer PAT-1 (adapted from http://sinterface.de/). (A) Macro-dosing system, (B) drop of the biological liquid, (C) light source, (D) camera lens, (E) an analog-digital converter, (F) computer system, (G) micro-dosing system and (H) thermostatic cell.

Hanging drop method is used for measuring the surface tension by PAT-1 device (“Topfen-Blasen-Profiltensiometer”) (Fig. 2). Its advantages include a small volume of liquid to be analyzed, a wide range of life-time measurements of the drop, that is, from 10 to 10,000 s (Zaitsev, 2016).

The experimental error of the measurement of surface tension by the method of hanging drop is about 0.1 mN/m. The main parameter of the droplet hanging on the capillary tip is its volume. The larger a volume of the drop, the more it is different from a spherical shape (Zaitsev, 2016).

#### Dynamic tensiometry parameters of cattle serum

**Figure 3:**
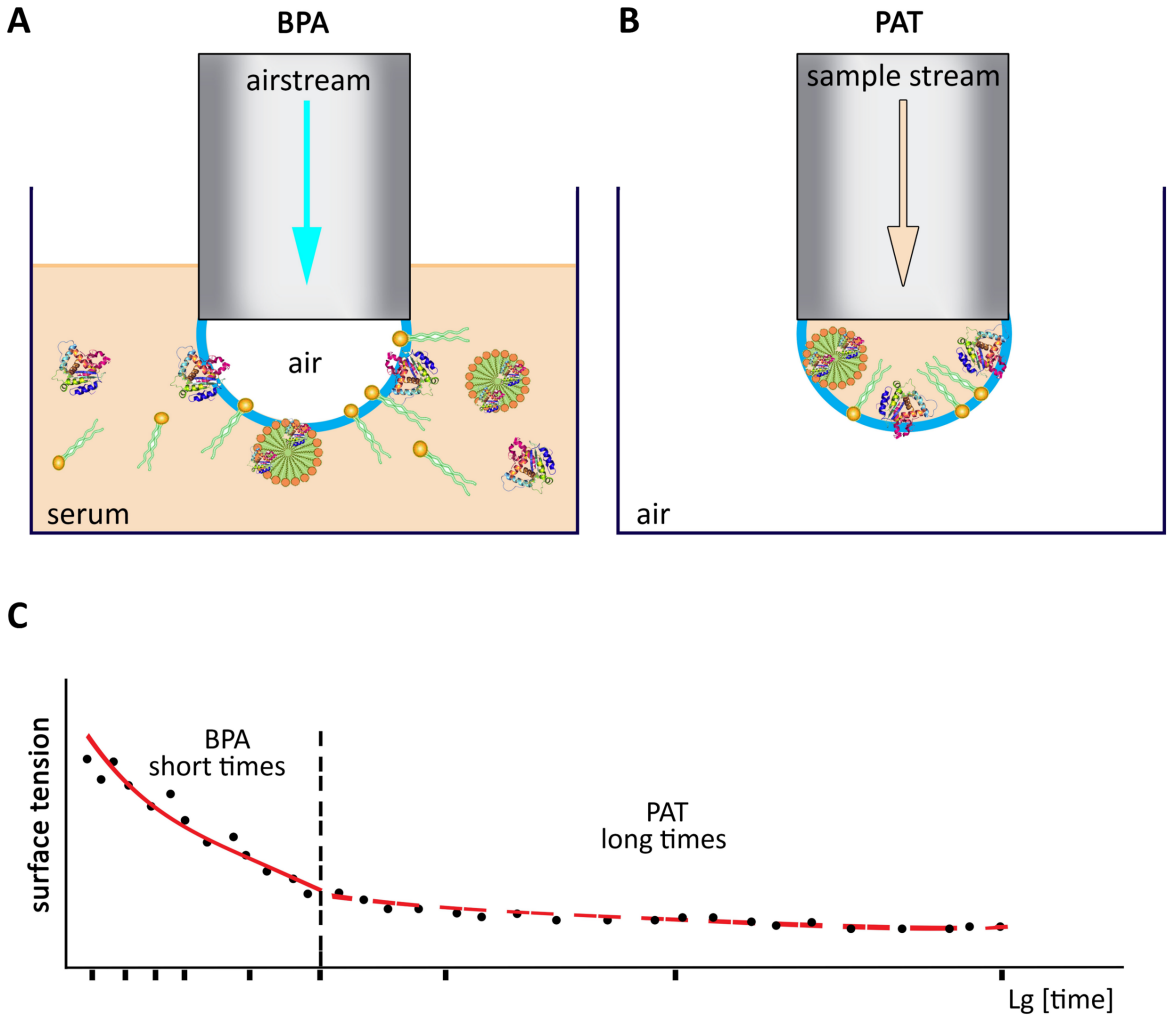
The general dependance of the DST parameters vs. time. (A) BPA method (short time range). (B) PAT method (long time range) of the DST measurements of the cattle blood serum. (C) The dependence of surface tension vs. lg[time] obtained by BPA and PAT methods.

The dependance of the dynamic surface tension parameters (Zaitsev, 2016) of the serum on the qualitative and quantitative composition has been described (Zarudnaya et al., 2010; Zaitsev et al., 2011b; Zaitsev, 2010, 2018). The dependance of the DT parameters (complete quantitative data set) of the serum of some animals (Fig. 3) have been obtained recently (Table 5) mainly by our group (Zarudnaya et al., 2010; Zaitsev et al., 2011a; Zaitsev, 2010, 2016, 2017) in contrast to some detached (single surface tension data) that has been described previously (Zaitsev, 2016; Voronina, 2017; Zaitsev, Fedorova & Maximov, 2019).

**Table 5:** The DT parameters of blood serum of cattle at various ages.

Parameters	Young calves, 6 month	Cows, 12 month	Cow, 18 month	Not pregnant cows	Second month of cow pregnancy	Sixth month of cow pregnancy
σ_0_, mN/m	72.07 ± 0.17	73.94 ± 0.49	73.34 ± 0.35	73.22 ± 0.31	74.39 ± 0.41	73.18 ± 0.31
σ_1_, mN/m	71.91 ± 0.43	73.52 ± 0.14	73.08 ± 0.48	72.86 ± 0.23	73.09 ± 0.51	72.45 ± 0.39
σ_2_, mN/m	66.80 ± 0.30	70.11 ± 0.42	69.86 ± 0.76	66.10 ± 0.48	64.24 ± 0.43	65.62 ± 0.37
σ_3_, mN/m	61.19 ± 0.66	64.35 ± 0.64	62.22 ± 0.55	60.45 ± 0.59	56.80 ± 0.59	58.97 ± 0.69
λ_0_, N∙m^−1^s^−1/2^	5.96 ± 0.15	3.16 ± 0.39	4.59 ± 0.39	6.07 ± 0.28	5.47 ± 0.36	7.33 ± 0.36
λ_1_, N∙m^−1^s^−1/2^	6.12 ± 0.23	5.70 ± 0.49	7.34 ± 0.38	6.79 ± 0.29	9.14 ± 0.35	7.98 ± 0.60

The DT values of the cattle serum (there were from 10 to 15 animals in each age-group) were reported (Zaitsev, 2016; Voronina, 2017). The dynamic tensiometry parameters were obtained from dependences of surface tension (σ) vs. time (*t*), so-called tensiogram (Fig. 3), at the particular points: *t* → 0 (σ_0_), *t* = 0.1 s (σ_1_), *t* = 1 s (σ_2_) and *t* = 10 s (σ_3_); or as the initial and final tilts of the tensiogram (λ_0_ and λ_1_ values, respectively).

All DT parameters, measured in cattle serum, undergo pronounced changes with animal age. For example, significant changes observed for the initial tilt of the tensiogram (λ_0_), that is, this value for 12 months animals decreased by 47% as compared to the heifers of 6 months age. In addition, the onset of 12–18 months of animal age led to adaptive changes in the parameters of surface tensions, so σ_0_–σ_3_ values increased or decreased by 3–5% as compared to the heifers of 6 month’s age (that in the frame of the experimental errors). By the time of physiological maturity (18 months), the values λ_0_ and λ_1_ are increased by 48% and 28%, respectively.

The onset of pregnancy is accompanied by numerous changes in the cow body, especially by the nervous system and endocrine glands. For example, a number of biologically active substances contained in the blood varies significantly during pregnancy, that leads to changes in the blood serum DT values. For cows at 2 or 6 months of pregnancy, the following major changes occurred: λ_1_ decreased by 24% or 15% and the value of λ_0_ increases by 20% or 25%, respectively, as compared to heifers of 18 month’s age. For not pregnant cows, the following major changes occurred: λ_1_ decreased by 35% and the value of λ_0_ increases by 10% as compared to heifers of 18 month’s age. In contrast to such significant changes in the λ_1_ and λ_0_ parameters, the changes in the σ_0_–σ_3_ values observed by 2–6% only, as compared with the pregnant cows (Table 5).

These data were obtained by MBP and hanging drop methods (using BPA-1P and PAT-1). The average equilibrium DT values (σ_∞_ at time → ∞) for male cattle were the following: young calves 46.92 ± 2.54 mN/m, at the middle age 47.03 ± 2.58 mN/m, adult bulls 46.62 ± 1.39 mN/m [81]. The average equilibrium DT values of the curve tilt (λ_∞_ at time → ∞) for male cattle were the following: young calves 0.31 ± 0.19 mN∙m^−1^s^−1/2^, middle aged 0.29 ± 0.12 mN∙m^−1^s^−1/2^, adult bulls 0.15 ± 0.05 mN∙m^−1^s^−1/2^ (Zaitsev, 2016).

Thus, there are significant changes in the cattle blood system occur due to age, sex, physiological state, which are “reflected” in the changes of the DT parameters.

#### Correlations between dynamic tensiometry and biochemical parameters of cattle serum

There are various correlations between DT and biochemical parameters of cattle serum are found recently (Zaitsev, 2016, 2017). A large amount of strong positive (21 items) and negative (13 items) or middle positive (7 items) and negative (5 items) correlations for heifer (6 months) were found (Table 6). The strong and middle (no matter positive or negative) correlations are promising for the further practical applications and only these correlations will be discussed below. There are positive correlations of σ_1_ with the level of proteins, triglycerides, cholesterol, glucose, calcium, sodium, and a negative correlation with the level of urea, potassium and chloride for heifers (Table 6). The σ_2_ values have a positive correlation with the level of albumin, triglycerides, cholesterol, glucose and sodium, and negative correlation with the level of total protein, urea, and chlorides. The σ_3_ value rises with increasing levels of urea, potassium and chloride, and decreases with increasing levels of albumin, triglycerides, cholesterol, glucose, calcium and sodium. The λ_0_ values have a positive correlation with majority of the biochemical parameters studied, whereas a negative correlation with urea, potassium and chlorides are found. The λ_1_ values have a positive correlation with the level of total protein, triglycerides, cholesterol, glucose, calcium, sodium, and a negative correlation with the level of urea, potassium and chloride (Table 6).

**Table 6:** Results of the correlation analysis for DT and biochemical parameters of blood serum of heifer (6 months).

Indices	σ_1_	σ_2_	σ_3_	λ_0_	λ_1_
Total protein, g/l	↑↑↑	↓↓	↓	↑↑	↑↑↑
Albumin, g/l	↑↑	↑↑↑	↓↓↓	↑↑	↓
Triglycerides, mM	↑↑↑	↑↑	↓↓↓	↑↑↑	↑↑↑
Cholesterol, mM	↑↑↑	↑↑	↓↓↓	↑↑↑	↑↑↑
Urea, mM	↓↓	↓↓↓	↑↑↑	↓↓↓	↓↓
Glucose, mM	↑↑↑	↑↑	↓↓↓	↑↑↑	↑↑↑
Calcium, mM	↑↑↑	↑	↓↓	↑↑↑	↑↑↑
Potassium, mM	↓↓↓	↑	↑↑↑	↓↓↓	↓↓↓
Sodium, mM	↑↑↑	↑↑	↓↓↓	↑↑↑	↑↑↑
Chlorides, mM	↓↓↓	↓↓	↑↑↑	↓↓↓	↓↓↓

**Notes:**

↑ (↓): weak positive (negative) correlation, the correlation coefficient below 0.3.

↑↑ (↓↓): middle positive (negative) correlation, the correlation coefficient of 0.3–0.69.

↑↑↑ (↓↓↓): strong positive (negative) correlation, the correlation coefficient over 0.69 (units: σ (mN/m), λ (mN∙m^−1^s^−1/2^)).

There are medium correlations between dynamic surface tension parameters and biochemical indicators of blood serum for heifer aged 1.5 years (Table 7). The tensiogram tilts rise with increasing concentration of total protein and total cholesterol in the serum, and decrease with increasing potassium concentration (Table 7). The σ_1_ value has a positive correlation with the level of sodium and inorganic phosphorus, and a negative correlation with the level of total protein, total cholesterol, and chlorides. The σ_3_ value has a negative correlation with the level of protein, lipids and chlorides, and a positive correlation with the level of urea, calcium and phosphorus. The λ_0_ value has a positive correlation with the levels of albumin, triglyceride, glucose and serum chlorides for heifers and negative correlation with the level of urea, total calcium, phosphorus and sodium. The λ_1_ value has a positive correlation with the level of cholesterol, urea, glucose, calcium and potassium, and a negative correlation with the level of total calcium and inorganic phosphorus (Table 7).

**Table 7:** Results of the correlation analysis for DT and biochemical parameters of blood serum of heifer (1.5 years).

Indices	σ_1_	σ_2_	σ_3_	λ_0_	λ_1_
Total protein, g/l	↓↓	↓↓	↓↓↓	↑↑	↑↑↑
Albumin, g/l	↑	↓↓	↓	↑	↑
Triglycerides, mM	↓↓	↓↓	↓	↑	↑↑
Cholesterol, mM	↓↓	↓↓	↓↓	↑	↑↑↑
Glucose, mM	↓↓	↓↓	↓↓	↑	↑
Urea, mM	↓	↑↑	↑↑	↑	↑
Total calcium, mM	↑↑	↑↑	↑	↓	↑
Phosphorus inorg., Mm	↑	↑↑	↑	↑	↑
Potassium, mM	↑↑	↑↑	↑↑↑	↓↓	↓↓
Sodium, mM	↑↑	↑	↑	↓	↓
Chlorides, mM	↓↓	↓	↓↓↓	↑	↑↑

Results of the correlation analysis of pregnant cow blood serum (Table 8) can be summarized as follows: the σ_1_ value has negative correlation with the level of albumin, triglycerides, cholesterol, glucose, and chlorides and a positive correlation with the level of calcium, phosphorus and potassium; the σ_2_ and σ_3_ parameters have a negative correlation with the level of total protein, lipid and chloride in the serum of lactating cows and a positive correlation with the level of urea, calcium and potassium (for σ_3_). The levels of urea, calcium, phosphorus and potassium have the greatest impact on the λ_0_ value. The λ_1_ value has a positive correlation with the level of protein, triglycerides, cholesterol, and chlorides and negative correlation with the level of urea and total calcium (Table 8).

**Table 8:** Results of the correlation analysis for DT and biochemical parameters of blood serum of pregnant cow.

Indices	σ_1_	σ_2_	σ_3_	λ_0_	λ_1_
Total protein, g/l	↓↓↓	↓↓	↓↓↓	↑↑	↑
Albumin, g/l	↑	↓↓	↓↓↓	↑↑	↑↑
Triglycerides, mM	↓	↓↓↓	↓↓	↑	↑↑
Cholesterol, mM	↓↓	↓	↓↓	↑↑↑	↑
Glucose, mM	↓	↓↓↓	↓	↑	↑↑
Urea, mM	↓	↑	↑↑	↓↓	↓
Total calcium, mM	↑	↑↑	↑↑	↓↓	↓↓↓
Phosphorus inorg., mM	↑↑	↑↑↑	↑↑	↓↓	↓↓
Potassium, mM	↑	↓↓	↓	↓	↓
Sodium, mM	↑↑	↓	↓	↓↓	↓
Chlorides, mM	↓↓	↓↓	↓↓	↑	↑↑

There is a positive correlation of σ_1_ with urea, chloride, albumin; and a negative correlation with the level of glucose (only σ_0_), calcium, cholesterol, total protein (only σ_1_), σ_3_ has a positive correlation with the level of glucose, calcium, cholesterol and negative correlation with the level of urea, chlorides, albumin for bovine (Table 9). The λ_0_ value has a positive correlation with the level of protein, urea, chloride, cholesterol and negative correlation with triglycerides, glucose and cations (potassium, sodium). There is a negative correlation for λ_1_ value (Table 9). Thus, the DT parameters depend on both quantitative and qualitative changes in the cow blood because of the particular physiological state (pregnancy, lactation) (Zarudnaya et al., 2010, Zaitsev, 2016, 2017; Voronina, 2017).

**Table 9:** Results of the correlation analysis for DT and biochemical parameters of blood serum of cows (3 years) during lactation (6 months).

Indices	σ_1_	σ_2_	σ_3_	λ_0_	λ_1_
Total protein, g/l	↓	↓↓	↓↓↓	↑	↑↑
Albumin, g/l	↓↓	↓↓↓	↓↓↓	↓	↑↑
Triglycerides, mM	↓↓	↓↓	↓↓	↑	↑↑
Cholesterol, mM	↓↓	↓↓	↓↓↓	↑	↑↑
Glucose, mM	↓↓	↓↓	↑	↑	↓
Urea, mM	↓	↑↑	↑↑	↓↓	↓↓
Total calcium, mM	↑↑↑	↑↑↑	↑↑	↓↓	↓↓
Phosphorus inorg., mM	↑↑	↓	↓	↓↓	↑
Potassium, mM	↑↑	↑	↑↑	↓↓	↑
Sodium, mM	↑	↑	↑	↓	↑
Chlorides, mM	↓↓	↓↓	↓↓	↑	↑↑

### Analysis of cell-free circulating nucleic acids

**Figure 4:**
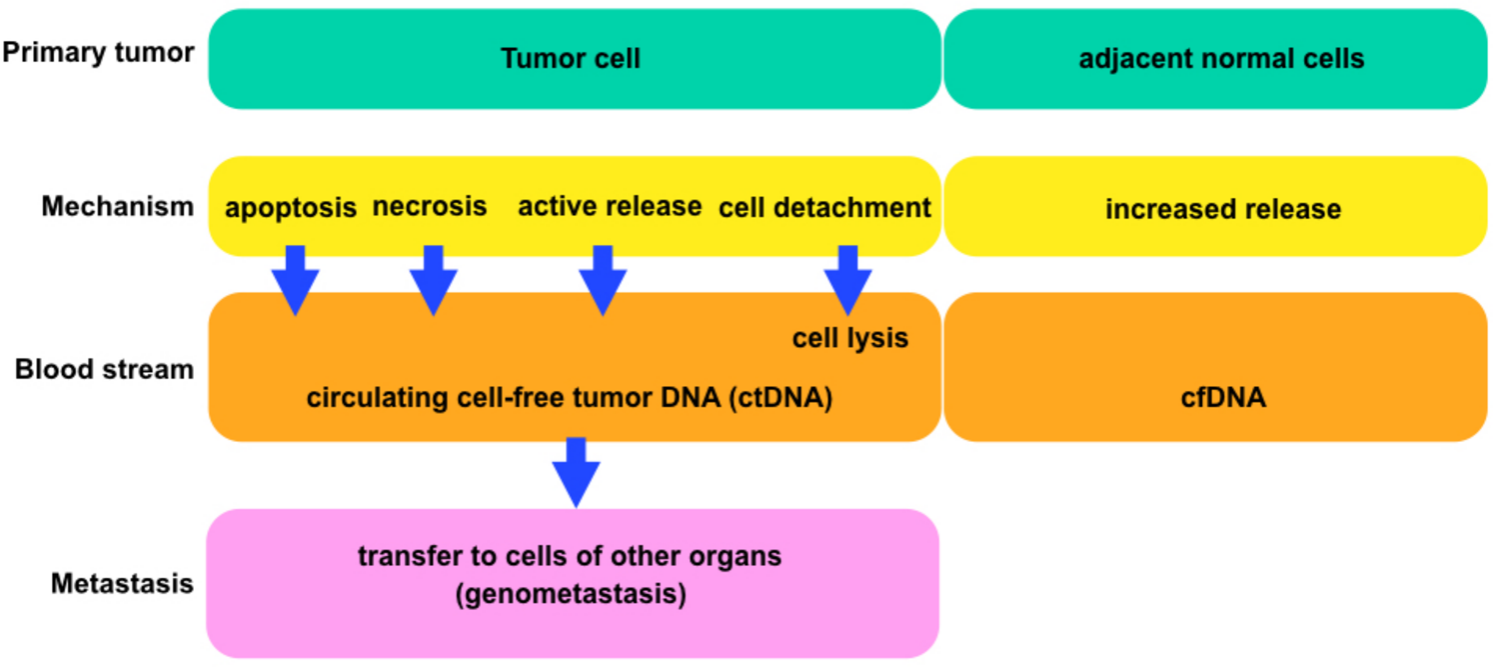
The general scheme comparing tumor and normal cells. Each block is showing the connections between cells and circulating tumor deoxyribonucleic acids (ctDNA) or cell-free deoxyribonucleic acids (cfDNA) (according to Jung, Fleischhacker & Rabien (2010)).

Circulating nucleic acids (Fig. 4) have been detected in plasma, serum and urine of healthy and diseased humans and animals (Fleischhacker & Schmidt, 2007). Both DNA and RNA can be isolated from serum and plasma (Beck et al., 2009; Brenig, Schütz & Urnovitz, 2002) and are commonly referred to cell-free circulating nucleic acids (cfDNA/RNA or CNA). Early work concentrated on detecting quantitative differences in circulating DNA (Brenig, Schütz & Urnovitz, 2002) between samples from patients with disease and samples from healthy individuals (Swaminathan & Butt, 2006). Although most of the data available in the literature (Beck et al., 2009; Brenig, Schütz & Urnovitz, 2002) on the possible diagnostic use of cfCNA were derived from studies of cancer patients, increases in circulating DNA have also been reported for other diseases, including trauma (Lo et al., 2000), stroke (Rainer et al., 2003), autoimmune diseases such as systemic lupus erythematosus (Li & Steinman, 1989) and diabetes mellitus (Lo et al., 2000).

In recent years advanced molecular analysis techniques have been applied to prenatal diagnosis based on circulating cell-free fetal DNA (ccffDNA), including quantitative PCR (qPCR), digital PCR (dPCR) and next-generation sequencing (NGS). There are different possible sources of fetal DNA entering maternal plasma, that is, direct transfer of DNA or placenta (the predominant source). Haematopoietic cells, for example, fetal erythroblasts, have previously been ruled out as potential origin and also cell-free fetal DNA in the amniotic fluid does not appear to be an origin (Lo, 2000). With these techniques noninvasive prenatal testing (NIPT) became available and allowed the detection of fetal sex (Perlado-Marina et al., 2013), fetal rhesus factor D (RhD) (Clausen, Damkjaer & Dziegiel, 2014), pregnancy-associated conditions (such as pre-eclampsia) (Rolnik et al., 2018), aneuploidies (Skrzypek & Hui, 2017), micro-deletions, micro-duplications, and the detection of paternally inherited monogenic disorders (Bustamante-Aragones et al., 2012; Hayward & Chitty, 2018) (Table 10). These methods also have the potential to be used for fetal whole-genome sequences and the detection of maternally inherited variants (Breveglieri et al., 2017; Hui et al., 2017; Perlado et al., 2016). In cattle (Table 10), identification of fetal sex and diagnosis of genetic diseases at an early stage can change the value of the pregnancy and give the chances to plan for better breeding. Bovine Y chromosome—specific sequences (i.e., SRY, TSPY) have been used for sex determination (Davoudi et al., 2012; Malarmathi et al., 2016; Tungwiwat et al., 2003). Sex has also been determined in cattle by simultaneous amplification of homologous nucleotide sequences on chromosomes X and Y, which differ in the length of the PCR products, for instance ZFX/Y (Kirkpatrick & Monson, 1993) and AMEL X/Y (Chen et al., 1999; Malarmathi et al., 2016). In addition, the diagnostic utility of CNAs has been shown in cattle and elk with bovine spongiform encephalopathy (Gordon et al., 2009). The analysis of cfDNA in domestic animals has been reported in several studies demonstrating cfDNA as useful markers in diagnosis (Beck et al., 2009, 2013; Brenig, Schütz & Urnovitz, 2002; Gordon et al., 2009; Mayer et al., 2013a, 2013b; Schütz et al., 2005).

**Table 10:** Diagnostic use of cfDNA in man and animals.

Trait/Application	Marker/Gene	Method	Species	References
Abortion diagnosis at early gestation	*DAZ4M8*	Real-time quantitative PCR	*Homo sapiens*	Stanghellini et al. (2006)
Fetal sex determination	*DYS14*	Real-time quantitative PCR; nested PCR	*Homo sapiens*	Chi, Kang & Hu (1999), Sekizawa & Saito (2001)
	*DYZ3*	PCR	*Homo sapiens*	Hahn et al. (2000)
	*DYZ1*	PCR	*Homo sapiens*	Zhao & Zou (2004)
	*SRY*	PCR	*Bos taurus*; *Ovis aries*; *Homo sapiens*	Kadivar et al. (2013), Malarmathi et al. (2016), Tungwiwat et al. (2003), Zhong, Holzgreve & Hahn (2000)
	*SRY* & *ATL1*	Nested PCR	*Homo sapiens*	Tungwiwat et al. (2003)
	*TSPY; Amelogenin, & BC1.2*	PCR, multiplex PCR	*Bos taurus*	Davoudi et al. (2012), Lemos et al. (2011)
	*BRY4a*	PCR	*Bos taurus*	Cenariu et al. (2012)
	*ZFX/Y*	Nested, allele-specific amplification; Microfluidics digital PCR	*Bos taurus*; *Homo sapiens*	(Kirkpatrick & Monson (1993), Lun et al. (2008a)
	*AMEL X/Y*	PCR	*Bos taurus*; *Ovis aries*; *Homo sapiens*	Asadpour et al. (2015), Chen et al. (1999), Trujillo-Tiebas et al. (2006)
X-linked disorders	rs6528633	Microfluidics digital PCR	*Homo sapiens*	Tsui et al. (2011)
Fetal Rhesus D Genotyping	*RHD*	PCR-based methods; digital PCR	*Homo sapiens*	Kolialexi, Tounta & Mavrou (2010), Scheffer et al. (2011), Sillence et al. (2017)
Aneuploidies	Polymorphic loci at chromosomes 13, 18, 21, X, and Y	Targeted sequencing	*Homo sapiens*	Nicolaides et al. (2013), Ryan et al. (2016)
	–	Massively parallel shotgun sequencing/whole genome sequencing	*Homo sapiens*	Palomaki et al. (2012), Taneja et al. (2016)
	–	Microarray-based digital analysis of selected regions	*Homo sapiens*	Juneau et al. (2014), Stokowski et al. (2015)
Monogenic diseases				
Cystic Fibrosis	*CFTR* mutations	Mutant enrichment with 3′-modified oligonucleotides qPCR; coamplification at lower denaturation temperature (COLD)-PCR coupled with Sanger sequencing; microarray	*Homo sapiens*	Galbiati et al. (2016), Guissart et al. (2015)
Beta-thalassemia and sickle-cell disease	SNPs along the β-*globin* gene cluster; mutations on *HBB*	Combined pyrophosphorolysis-activated polymerization and melting curve analysis; COLD-PCR coupled with Sanger sequencing; microarray; Digital relative mutation dosage analysis; Taqman genotyping assays; MALDI-TOF mass spectrometry	*Homo sapiens*	Breveglieri et al. (2017), Galbiati et al. (2016), Li & Makrigiorgos (2009), Lun et al. (2008b), Phylipsen et al. (2012)
Neurofibromatosis type 1	*NF1* mutations	Droplet digital PCR	*Homo sapiens*	Gruber et al. (2018)
Transmissible spongiform encephalopathies	Polymorphisms in repetitive genomic nucleic acid sequences	Mass sequencing approach	*Bos taurus; Cervus elaphus*	Beck et al. (2009), Gordon et al. (2009), Schütz et al. (2005)

However, not only the detection of DNA is promising in noninvasive diagnosis in animals. Although RNA is very labile and easily degraded, it has been shown that both endogenous and exogenous circulating RNA in blood, including microRNAs, small and long non-coding RNAs can be detected and used for diagnosis (Fleischhacker & Schmidt, 2007; Swaminathan & Butt, 2006). A rather novel approach is the use of proteomics in minimal-invasive diagnosis. A recent publication showed that 24 plasma proteins can be used for the determination of beef tenderness (Boudon, Henry-Berger & Cassar-Malek, 2020).

## Conclusions

Thus, in addition to standard methods recent technologies such as dynamic tensiometry of blood plasma (serum) and PCR analysis of particular markers are in progress. Interpretation of major biochemical parameters usually is similar across major animal species, but there are a few peculiarities for cattle. The DT parameters depend on both quantitative and qualitative changes in the cow blood because of the particular physiological state (pregnancy, lactation). The numerous correlations between DT data and biochemical parameters of cattle serum have been obtained and discussed. The revealed correlations between DT and biochemical parameters of cattle serum tcan simplify the procedure and to speed up the final diagnosis decision.

Changes in the cell-free nucleic acids circulating in the blood have been studied and analyzed in a variety of conditions, for example, pregnancy, infectious and chronic diseases, and cancer. CfDNA can easily be detected using standard molecular biological techniques like DNA amplification and next-generation sequencing. The application of digital PCR even allows exact quantification of copy number variations which are for example important in prenatal diagnosis of chromosomal aberrations.

## Abbreviations

BAS: biologically active substances
ccffDNA: circulating cell-free fetal DNA
cfDNA: cell-free deoxyribonucleic acids
ctDNA: circulating tumor deoxyribonucleic acids
DST: dynamic surface tension
DT: dynamic tensiometry
MBP: major biochemical parameters
MPS: massively parallel sequencing
NIPD: noninvasive pre-natal diagnosis
NGS: next-generation sequencing
PCR: polymerase chain reaction
dPCR: digital PCR
qPCR: including quantitative PCR
RBC: red blood cell
ST: surface tension
WBC: white blood cell

## Additional Information and Declarations

### Data Availability

The following information was supplied regarding data availability: This is literature review and there is no raw data.
